# Tumor-derived exosomes in colorectal cancer progression and their clinical applications

**DOI:** 10.18632/oncotarget.20117

**Published:** 2017-08-10

**Authors:** Jianbiao Zhou, Xiao-Lan Li, Zhi-Rong Chen, Wee-Joo Chng

**Affiliations:** ^1^ Cancer Science Institute of Singapore, National University of Singapore, Centre for Translational Medicine, Singapore 117599, Republic of Singapore; ^2^ Department of Medicine, Yong Loo Lin School of Medicine, National University of Singapore, Singapore 117597, Republic of Singapore; ^3^ Department of Gastroenterology, Suzhou Municipal Hospital (Eastern), Suzhou Hospital Affiliated to Nanjing Medical University, Suzhou, 215001, China; ^4^ Department of Hematology-Oncology, National University Cancer Institute of Singapore (NCIS), The National University Health System (NUHS), Singapore 119228, Republic of Singapore

**Keywords:** exosomes, colorectal cancer (CRC), biomarker, cancer therapy, microRNA (miRNA)

## Abstract

Colorectal cancer (CRC) ranks as the third leading cause of cancer mortality in both of men and women worldwide due to its metastatic properties and resistance to current treatment. Recent studies have shown that tumor-derived exosomes play emerging roles in the development of cancer. Exosomes are nano-sized extracellular vesicles (EVs) that contain lipids, proteins, DNAs, and RNA species (mRNA, miRNA, long non-coding RNA). These exosomal cargos can be transferred locally and systemically, after taken by recipient cells, so exosomes represent a new form of intercellular communication. There is increasing evidence demonstrating that exosomes control a wide range of pathways bolstering tumor development, metastasis and drug resistance. This review provides an in-depth and timely summary of the role of exosomes in CRC. We first describe the common features and biogenesis of exosomes. We then highlight important findings that support the emerging roles of exosomes in CRC cell growth, invasion and metastasis, as well as resistance to treatment. Finally, we discuss the clinical application of exosomes as diagnostic biomarkers, *in vivo* drug delivery system and the potential of novel exosome-based immunotherapy for CRC.

## INTRODUCTION

EVs (extracellular vesicles), derived from various cells transfer information efficiently to recipient cells locally or systemically, thus play an important role in numerous biological activities [1]. EVs are divided into three subsets: exosomes, microvesicles, and apoptotic bodies depending on their size [2], and exosomes, in particular, are of great interests among the cancer research community. Exosomal genetic materials that are composed of proteins, messenger RNAs, microRNAs (miRNAs), long non-coding RNAs (lncRNAs), DNAs, lipids and other small molecules, can change the biological behaviors of target cells significantly after being taken in. As such, exosomes represent a distinct type of cell-to-cell communication. There is increasing evidence demonstrating the important role of exosomes in tumor development and drug resistance [3]. Colorectal cancer (CRC) is a disastrous disease with high prevalence and low 5-year survival, especially in Stage IV metastatic CRC [4], thereby the urgent need to explore more sensitive diagnostic methods and more efficient treatment. With the close association between exosomes and cancer, more researchers are focusing on the specific function of exosomes in colorectal cancer. In this review, we summarize the current knowledge on exosomes in CRC, supporting a key role of exosomes in CRC pathogenesis, biomarkers and therapeutic applications.

### Exosomes and their biological functions

Exosomes of 30–100 nm in diameter, one of the EVs subsets, were first reported as a pattern of cell membrane rollover in sheep reticulocyte maturation [5, 6]. Since then, a significant volume of research have uncovered the novel functions of exosomes and provided insight into their structures, thus redefining the exosomes field [7, 8]. Exosomes can be identified in almost all human body fluids, such as blood, urine, saliva, and pathological ascites [9–12]. About 2,000 trillion exosomes have been detected in normal human blood and about 4,000 trillion in blood of cancer patients. In general, cancer cells would generate more exosomes than their normal counterparts [13]. Exosomes are generated in a unique manner from multi-vesicular endosomes (MVEs), also known as multi-vesicular bodies (MVBs), which are components of the endocytic pathway. The sizes of MVBs range from 250 to 1000 nm in diameter. The formation of MVEs start from a portion of the limiting membrane of an endosome, invaginating and budding into its own lumen. There are intraluminal vesicles (ILVs) with size of 30 to 100 nM in diameter. If MVBs fuse with lysosomes, its initiates the degradation of ILVs and their contents by lysosomal hydrolases. If MVBs fuse with plasma membrane, ILVs are released into the extracellular space and these released ILVs are defined as exosomes. ESCRT (endosomal sorting complexes required for transport) complexes, sphingolipid ceramide and Rab GTPase (guanosine triphosphate) family (such as Rab11 and Rab27) are involved in the biosynthetic process as key regulatory factors [14–16]. So, some of markers associated with ESCRT are conserved in exosomes secreted by different types of cells, such as Alix, CD63, CD81, TSG101. However, these makers are not exclusive to exosomes, because they also appear in the vesicles derived from membrane shedding.

Emerging evidence indicate that exosomes provide a means for cell-to-cell communication by transporting their cargo and delivering it to target cells locally or remotely. Metaphorically, exosomes are the ships or boats that transfer the cargos, which include DNAs, mRNAs, miRNAs, IncRNAs, proteins, lipids and other metabolites that have biological activities. Notably, the lipid bilayer membrane of exosomes effectively provide their cargos from degradation by separating from the extracellular environment.

**Figure 1:**
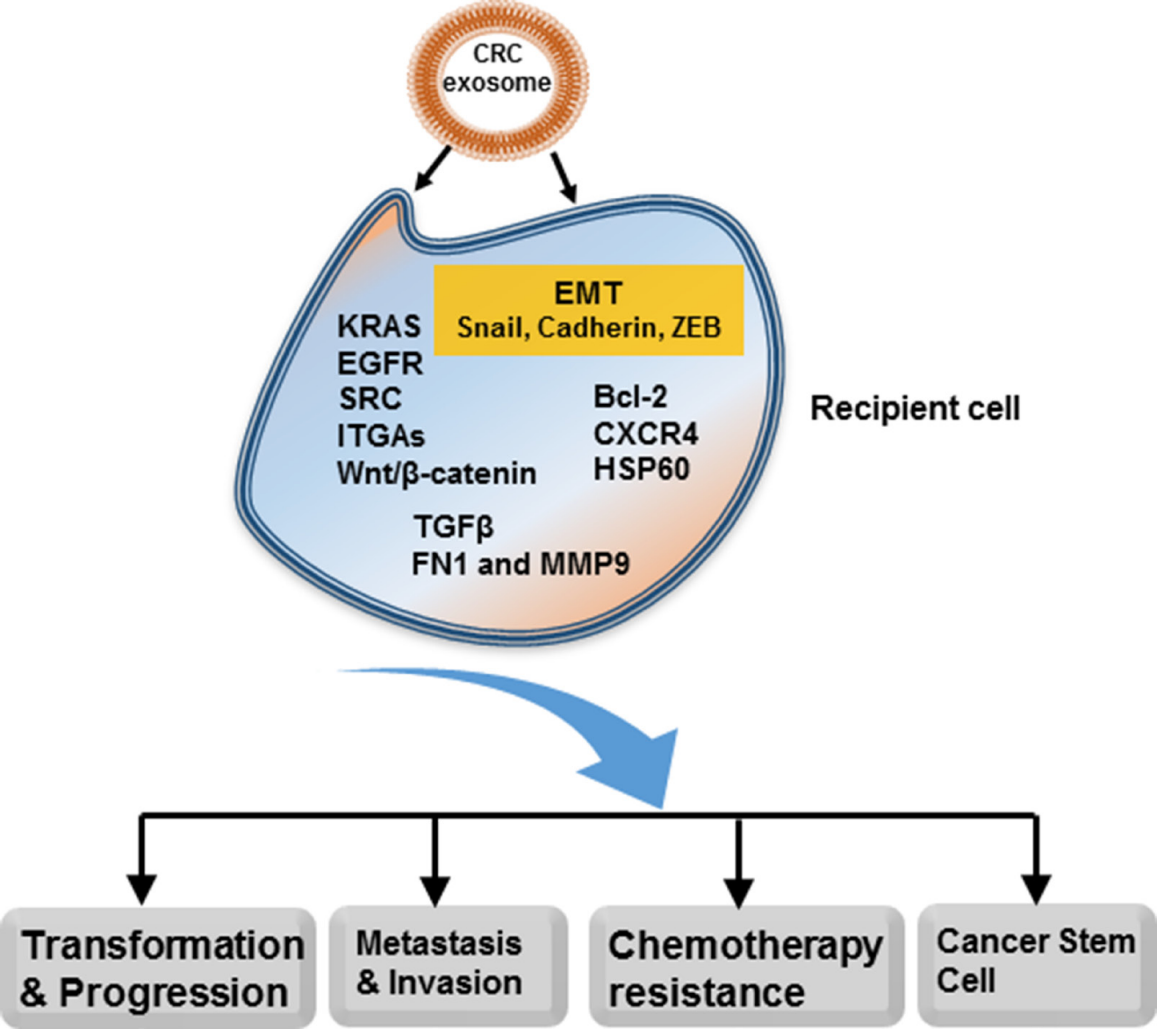
Multiple roles of exosomes in CRC Exosomes can activate critical oncogenic signaling pathways in the recipient cells (cancer cells or niche cells) to promote tumor transformation, progression, invasion and metastasis. Exosomes are also involved in formation of cancer stem cells and development of drug resistance. CRC-derived exosomes can modulate biological functions of recipient cells through locally and systemically transferring proteins, DNAs, mRNAs, miRNAs, lncRNAs, lipids, etc.

The biological behaviors of exosomes are heterogeneous, which could be due to their diverse secretory cell types with different cell status, for instance, endothelial cells, epithelial cells, fibroblasts, neurons, immune cells, and mesenchymal stem cells [13, 17–19]. Through the circulation, exosomes act on target cells, fusing with plasma membrane and releasing contents to exert specific functions. Or, these cargoes can be locally transferred into neighbor cells within the same environment. Great efforts have been made to investigate the distinct mechanisms of exosomes participating in tumorigenesis and progression via various signaling pathways. It has been proven that exosomal contents modify tumor microenvironment, facilitate EMT (epithelial-to-mesenchymal transition) and cytoskeleton reorganization, promote tumor angiogenesis, and influence tumor immunity, resulting in CRC formation, invasion and metastasis, and therefore have important clinical relevance (Figure 1).

### Exosomes in the transformation of CRC

Sporadic CRC is characterized by a multi-step transformation of normal colonic mucosa to adenocarcinoma in individuals without genetic predisposition or family history of CRC. After exposure to cancer cell-derived exosomes, epithelial cells with non-malignant phenotype received exosomal cargos, such as oncogenic mRNA or miRNA, and pro-angiogenic proteins, resulting in an altered biological property for tumor initiation [20, 21]. This pioneering work in breast cancer, and glioblastoma indicate that some tumor-derived exosomes are capable of enabling the transformation of normal cells into malignant states. Similarly, it has been shown that exosomes secreted from human CRC cells transfer mRNAs, miRNAs and natural antisense RNAs (asRNAs) into liver cancer and lung cancer cells [1]. The V-Ki-ras2 Kirsten rat sarcoma viral oncogene homolog (KRAS) is a prominent oncogene that encodes a small GTP-binding protein called KRAS. KRAS mutations, mainly in codon 12 or 13, occur in 35 to 45 % of patients with CRC, and are frequently associated with lung and brain metastasis [22]. Two independent studies have found compelling evidence that mutant KRAS gene influence the compositions and functions of exosomes produced by CRC cells [23, 24]. Proteomic analysis of exosomes secreted by isogenic KRAS wild-type or mutant (G13D) CRC cell line revealed that the composition of the exosomes differ dramatically [24]. Many tumor-promoting proteins, including KRAS, EGFR, SRC family kinases, and integrins are specifically enriched in exosomes from mutant KRAS cells. Importantly, when exosomes released by mutant KRAS cells transfer mutant KRAS protein into these KRAS wide type non-transformed cells, their growth in three-dimensional culture is promoted [24]. The second study examined the global small RNA profiles from exosomes isolated from isogenic CRC cell lines which only differ in their KRAS status [23]. A mutant KRAS-specific pattern of secreted miRNAs was observed, indicating KRAS-dependent biogenesis or sorting of exosomal miRNAs. Specifically, miR-10b was selectively increased in KRAS wild-type exosomes, while miR-100 was particularly heightened in mutant exosomes. Notably, miR-100 released by mutant KRAS CRC cells could further amplify miR-100 function in recipient wild-type cells as evidenced by the decreased expression of some target genes of miR-100 [23]. Collectively, these findings are important because they provide novel insight of the contributions of mutant KRAS exosomes to CRC. Therefore, targeting exosome either by inhibiting its production or by blocking its transfer should be exploited in the development of novel treatments for patients with KRAS mutation who are at higher risk of developing metastatic malignancies. A previous study has attempted to detect the mRNA expression with exosome-related markers in healthy subjects, colorectal adenoma and carcinoma patients [25]. At the *in situ* protein level, ALIX (ALG 2-interacting protein X) expression is significantly reduced in adenoma and carcinoma patients compared with healthy persons. ALIX-positive particles including MVB-like structures were found in carcinoma and the surrounding microenvironment of tumor nest. It is therefore suggested that the gradual transition of ALIX, which takes part in multi-vesicular body (MVB) and exosome formation, facilitates the colorectal adenoma-carcinoma progression.

It is now established that exosomes are crucial components of the tumor microenvironment. Cancer cell-derived exosomes are able to stimulate fibroblast differentiation into tumour-promoting stromal myofibroblasts through a TGFβ1-dependent pathway, accelerating angiogenesis and tumor growth [26]. The host microenvironment in which the CRC cells reside is hypoxic, due to increased oxygen consumption as a result of hyperplasia and decreased oxygen delivery. This hypoxic niche protects CRC cells from chemotherapy and attack from immune cells. Under hypoxic conditions, Wnt4 is particularly enriched in exosomes secreted by CRC cells and increases β-catenin nuclear translocation in endothelial cells depending on the transcription factor HIF1α. The hyper-activation of Wnt/β-catenin signaling by CRC cell-derived exosomes enhances the proliferation and migration of endothelial cells, promoting tumor growth and angiogenesis *in vivo, in* animal study [27].

In this regard, CRC cells produce several types of exosomes, which promote the growth and transformation of CRC by influencing CRC cells or the reprograming of microenvironment to support CRC cell proliferation.

### Regulation of CRC invasion and metastasis by exosomes

The 5-year survival rate of CRC patients with distant metastases is extremely poor, less than 10%. Furthermore, approximate 25% of newly diagnosed CRC patients have distant metastases at presentation [28, 29]. In addition, one in four patients who have localized CRC would develop distant metastases within two years after diagnosis. Therefore, metastasis is an important challenge and the leading cause of death in CRC.

CRC has a characteristic metastatic pattern in which distant metastases appear mainly in the liver and less commonly in the lung, bones and brain [30, 31]. The phenomenon that some types of cancer cells preferentially home and colonize to specific organs is defined as organ tropism. Different type of metastatic cancer cells vary remarkably in organ tropism [32]. For example, pancreatic ductal adenocarcinoma (PDAC) is highly metastatic to liver [33]. Although the pattern of organ-specific metastasis is clear, the molecular programs that contribute to this tropism has not been well understood. Recent studies on tumor exosomes shed some light on this longstanding mystery of organotropism for cancer metastatic [34, 35]. Recent reports from the Lyden’s laboratory showed that exosomes isolated from lung-, liver- and brain-tropic tumor cells fuse preferentially with resident cells respectively at their predicted metastatic sites [34]. Proteomic analysis of these exosome populations uncovered that unique intergrin expression patterns are the determining factors for this organotropism. Specifically, lung-tropic exosomes express intergrins a_6_β_4_ and a_6_β_1_, while liver-tropic exosomes express intergrins α_v_β_5_. Taken together, the main function of exosomes in promoting metastasis is to prepare pre-metastatic niche in specific distant organs through the transfer of intergrins or macrophage migration inhibitory factor (MIF) into receipt cells [34, 35].

In addition to its important roles in the transformation and growth of CRC cells, stromal or cancer cell-derived exosomes have been proven to induce cancer invasion and metastasis. Data from *in vitro* and *in vivo* studies have shown that malignant cells enhance migration behavior and metastatic property of benign cells through cell-to-cell communication by virtue of transferred exosomes to promote tumor progression [36]. When exposed to primary or metastatic CRC-derived exosomes, MSCs (mesenchymal stromal cells) derived from CRC masses not only formed umbilicated spheroids generally observed in the core of growing tumor, but also appeared to undergo functional change with higher proliferation, migration, and invasion [37].

Three recent studies revealed a list of specially expressed miRNAs transferred by exosomes that are involved in proliferation and migration of CRC cells (summarized in Table 1). miR-210 has been known to contribute to EMT, anoikis resistance and metastatic potential in cancers. The expression of miRNA-210 was markedly upregulated in exosomes as compared with its intracellular level in HCT8 cells, indicating its role in maintaining the growth-permissive microenvironment for primary cancer cells and guiding the new location for metastatic cells [38]. The process of EMT refers to the phenomenon in which adhesive, non-mobile epithelial-like cells transform into mesenchymal-like cells, gaining the ability to migrate to distant anatomical sites [39]. Studies in the last decade have documented the essential roles of EMT in the formation of cancer metastasis. One key step in the event of EMT is the loss of E-cadherin and the transcription factors (TFs) which repress E-cadherin, known as EMT-inducing TFs, which includes SNAI, ZEB1, ZEB2, E47 and KLF8. Recent research suggests the involvement of exosomes in the induction of EMT, attributing to the metastatic progression of CRC [40]. Exosomal miR-200c, miR-141 and miR-429 from naïve CRC CCL227 cells decreased expressions of ZEB2 and SNAI with the role of direct transcriptional repressors in CRC metastasis. In addition, these exosomal miRNAs decelerated CCIDs (circular chemorepellent-induced defects) formation in blood endothelial cell (BEC)-barrier as well as LEC (lymphatic endothelial cell)-barrier which are the gates of tumor migration via blood vessels and lymphatics [41].

**Table 1:** Summary of major exosomal miRNAs and their functional roles in CRC

References	Exosomal miRNAs	Major Conclusions
[38]	miR-210	Contributing to EMT, anoikis resistance and metastatic potential
[83]	miR-220C, miR-141	Up-regulating EMT, promoting invasion
[42]	miR-375	Inducing CRC cell apoptosis through blocking Bcl-2
[41]	miR-200c, miR-141, miR-429	Decreasing expressions of ZEB2 and SNAI, reprogramming the stroma in the metastatic process
[61]	let-7a, miR-1229, miR-1246, miR-150, miR-21, miR-223, and miR-23a	The serum exosomal level of these 7 miRNAs are significantly higher in CRC patients than healthy controls.
[62]	miRNA-19a	A prognostic biomarker for recurrence in CRC
[63]	miR-379	Inhibiting CRC cells proliferation and migration
[64]	miR-193a	Higher levels of exosomal miR-193a are found in advanced stage patients.
[23]	miR-100	miR-100 is selectively enriched in exosomes derived from mutant K-Ras CRC cells and transferred into wild type CRC cells.

In contrast to the function of the above-mentioned miRNAs as oncogenes, miRNA-375 has been identified as a tumor suppressor gene in CRC. Exosomes isolated from liver metastasis of CRC carrying miR-375 affect CRC cell apoptosis through the Bcl-2 pathway [42]. Bcl-2 was down-regulated in HCT116 cells transfected with the miR-375 mimic, promoting apoptosis. On the contrary, Bcl-2 was up-regulated after HCT116 cells were treated with miR-375 inhibitor, increasing cell proliferation. The liver metastatic CRC cell line (HT-29)-derived exosomes increased CXCR4 expression in the metastatic microenvironment further to promote cancer metastasis [43]. A 3D (three-dimensional) model of CRC cells was established to study the mechanisms by which CRC-triggered LEC (lymphatic endothelial cell) and BEC (blood endothelial cell) barriers are breached.

In addition to miRNAs, protein components in the exosomes also have been systematically investigated for their role in the pathogenesis of CRC metastasis Proteome profiling was conducted to compare the protein cargo of exosomes released from two isogenic human colorectal cancer cell lines: one primary cell line and its metastatic variant [44]. This study revealed that metastatic CRC cell exosomes selectively enrich some key metastatic factors and signal transduction molecules compared with primary CRC cell exosomes. Importantly, MET signaling complex, TNIK-RAP2A complex are uniquely expressed in metastatic CRC cell exosomes [44].

Taken together, these observations reveal that exosomes not only determine the site-specific metastasis, but also mediate intercellular communication to stimulate the process of EMT, migration and invasion of CRC cells.

### Exosomes induce chemotherapy resistance in CRC

Currently, chemotherapy remains an important remedy for advanced CRC [45] . However, a large number of patients show various degrees of drug resistance that directly resulted in poor prognosis [46]. The molecular pathways associated with drug resistance have been extensively studied over the last few decades, including overexpression of ATP-binding cassette (ABC) efflux transporters, p53 mutation, and deregulation of apoptotic pathways or DNA repair pathways. Despite these understandings, CRC remains one of the leading cause of cancer death worldwide, mainly due to metastasis and treatment resistance. Many studies have highlighted the relevance of exosomal miRNAs as drug-resistance mediators, revealing previously unrecognized mechanism [47–49]. Cancer cell secreted-exosomes transfer a variety of miRNAs carrying resistant clues into recipient cells and convert them to drug resistant phenotypes by targeting genes involved in the regulation of cell cycles and apoptosis. Moreover, the roles of exosomes in chemoresistance also arise from their ability to remove chemotherapeutic agents from cancer cells or preventing their entry into the nucleus. These novel drug resistance mechanisms have been confirmed in breast cancer, ovarian cancer, melanoma, and other types of solid tumors [50–52].

More than 10 years ago, cancer stem cell (CSC) has been described as the origin of various cancers and is associated to disease progression [53]. These CSC populations are rare, and are functionally and phenotypically different form the bulk of tumor cells. CSCs are not only resistant to the conventional chemotherapy, but also are more enriched after chemotherapy [54]. Importantly, CSCs acquire self-renewal function and are poised to propagate, leading to therapeutic failure and cancer recurrence [55, 56]. Recent studies also proved the involvement of exosomes in CSC formation and resistance to cell death caused by chemotherapy both *in vitro* and *in vivo* [57, 58]. Fibroblasts-derived exosomes trigger spheroid formation and tumorigenic ability of CSCs through the Wnt signaling pathway, thus enhancing drug resistance [58]. The miRNA-200 family members released from exosomes were confirmed to suppress EMT–regulating transcription factors. The absence of miR-200c, miR-141 and miR-429 in 5-fluorouraclil-resistant colon cancer cells sensitized LECs (lymphendothelial cells) to the migratory signal and accelerated CCIDs formation in BEC-barrier [41, 59].

### Clinical potential of exosomes in CRC patients

Given the important roles of exosomes in tumor formation, invasion and metastasis, and chemoresistance, exosomes have now been considered as an excellent source of diagnostic markers or therapeutic targets for CRC patients.

### Novel biomarkers

Early studies quantifying the total amount of exosomes in plasma samples using flow cytometry have demonstrated that the fraction of exosomes in CRC patients were more than two-fold higher than in healthy controls [60]. Furthermore, elevated levels of exosomes in plasma of patients have been associated with high levels of cancer antigen and with poorly differentiated tumors. CRC patients with high amount of plasma exosomes survived shorter than those with low levels [60]. miRNAs encapsulated in exosomes are typically transported to the circulation to modulate target cells, thereby playing an important role in CRC progression. Many investigations have confirmed the abnormal expression level of particular miRNAs in body fluids of CRC patient as reliable biomarkers for early diagnosis and staging assessment. A panel of seven exosomal miRNAs, including let-7a, miR-1229, miR-1246, miR-150, miR-21, miR-223, and miR-23a, are found to be expressed significantly higher in the serum of primary CRC patients, even in those with early stage disease, than in healthy controls. Importantly, their levels decreased significantly after surgical resection of tumors [61]. In addition, the difference in expression of diverse miRNAs derived from exosomes in CRC patients’ serum before and after therapy is the basis for evaluation of therapeutic effect and prognosis. For example, exosomal miR-17-92a cluster expression level in serum correlates with the recurrence of CRC. This case-control study also reported that patients after surgery with high level of serum exosomal miRNA-19a exhibited poor prognosis in contrast to those with low expression [62]. Clancy and coworkers engineered HCT116 cells to express miR-379 within exosomes. These cells exhibit significantly depressed cells proliferation and migration [63]. Recently, a novel mechanism by which exosome indirectly contribute to progression of CRC was reported [64]. Tumor-suppressive miR-193a was selectively sorted out form cancer cells into exosome in a major vault protein (MVP)-dependent manner. Hence, the level of circulating exosomal miR-193a has proved to be higher in CRC patients with more advanced disease compared with those with early stage of CRC and healthy controls [64].

CircRNAs (Circular RNAs), which are more abundant in exosomes than in the parental cells, participate in the process of cancer progression. CircRNAs from colorectal cancer cells with different KRAS status were analyzed. The result showed the down-regulation of circular RNAs in KRAS mutant DLD-1, DKO-1 and HCT116 cells compared with CRC cells carrying wild-type KRAS (DKs-8 and HKe3) [65]. This evidence suggests that circRNAs could be used as a class of new biomarkers for screening CRC patients with mutant KRAS gene for risk and prognostic assessment.

With the advance in mass spectrometry-based proteomic tools, coupled with improved purification methods for exosomes, the proteomic cataloguing of exosomes from diverse cancer types has revealed some common membrane and cytosolic proteins, as well as a set of proteins specific for different type of cancers, reflecting the original host cell. Chen and colleagues discovered that 36 proteins were upregulated and 22 proteins were downregulated in the serum-purified exosomes (SPEs) of CRC patients as compared to normal volunteers [66]. Pathway and network analysis revealed two interaction hubs centered on the proteins FN1 and matrix metalloproteinase-9 (MMP9), which are upregulated in the SPEs of CRC patients and pathways that are involved in cytoskeletal organization and integrin signaling in tumor progression and metastasis by reprogramming tumor microenvironment [66]. It has been consistently validated that heat shock protein 60 (Hsp60) plays a pivotal role in tumorigenesis. Hsp60 is found to be increased in exosomes liberated by tumor cells and circulated in the blood system. Interestingly, exosomal and circulating Hsp60 soon return to normal level after surgical resection of CRC tumor [67]. Therefore, exosomal Hsp60 could serve as a novel biomarker for the diagnosis of CRC and estimate of cancer treatment.

### New strategies for anticancer therapy

The remarkable success in translating basic science into survival in recent years is adoptive cell therapies with chimeric antigen receptor (CAR) engineered T cells (CAR-T) and immune checkpoint inhibitors in certain types of hematologic malignancies and solid tumors [68]. Dendritic cells (DCs) are potent antigen-presenting cells (APCs), originating from a common progenitor, the monocyte and dendritic cell progenitor (MDP) in bone marrow [69]. DCs play a key role in regulating both innate immune response and adaptive immune response [70].

Thus, DCs as “Nature’s adjuvant” have been tested in numerous clinical trials as cellular mediators for therapeutic vaccination of patients with cancer [71]. Soon after the discovery of exosome, researchers demonstrated that exosomes secreted by DCs, also known as dendritic cell–derived exosomes (Dex), can modulate immune response against cancer [72]. Pioneer work using immune-electron microscopy and proteomic technique revealed that Dex contain abundant MHC class I, MHC class II molecules, CD63, CD81, intergrins, milk fat globule-EGF-factor VIII [MFG-E8]), and other membrane proteins, displaying strong immunostimulatory characteristics [73, 74]. In a preclinical mouse model, exosomes derived from tumor peptide-loaded DCs demonstrated better anti-tumor potency than tumor peptide-loaded DCs themselves, eliciting tumor-specific cytotoxic T lymphocytes (CTLs) response [73]. The first two clinical trials using autologous Dex loaded with the MAGE tumor antigens in patients with non-small cell lung cancer (NSCLC) or melanoma in 2005 reported that Dex therapy was safe and activation of immune effectors was observed [75, 76].

After these two initial trials, the feasibility and effectiveness of Dex-based immunotherapy have been explored in other types of cancers too, including CRC. It is important that exosomes trigger anti-tumor immunity [77]. Several studies have demonstrated that exosomes secreted from cells such as dendritic cells (DCs), mast cells and activated T and B cells regulate the adaptive immunity [78, 79]. Cancer cell-derived exosomes not only inhibit natural killer (NK) cell proliferation and cytotoxic effect but also induces T cell apoptosis by carrying Fas ligand [80, 81]. DC-derived exosomes with T-cell-dependent antitumor effect have been evolving to a phase II trial in France. In China, phase I clinical trial for advanced CRC has been performed with ascites-derived exosomes (Aex) and GM-CSF (granulocyte-macrophage colony-stimulating factor). The combination of Aex and GM-CSF efficiently induces antitumor cytotoxic T lymphocyte response as a safe and viable immunotherapy of advanced CRC [82]. In short, DC-mediated immunotherapy combined with GM-CSF or chemotherapy produces longer survival benefit in CRC patients, especially at the metastatic stage. Overall, Dex has proven to be safe and feasible, however, its clinical success was only limited in a few small trials. To achieve broader and sustainable clinical response, more research should focus on engineering exosome to elicit stronger anticancer immunogenicity. Given the remarkable advance in bioengineering and oncoimmunology, exosome-based immunotherapy alone or in combination with other established therapeutics could be one of the important ammunition to fight against cancer in the future.

In addition to engineering miRNA into exosomes, it should also be noted that exosomes could be used as vector in the delivery of chemotherapeutic drugs, replacing liposomes. Exosomal delivery system has the advantages of slower clearance from the bloodstream, thus increasing effectiveness of delivery, as well as higher specificity for CRC cells.

### Future perspectives

Because of their small size and diverse population, the precise mechanisms of exosomes biogenesis and their multiple impacts on CRC cells and tumor microenvironment through paracrine, autocrine and telecrine routes are not well-defined and need to be further delineated. Accumulating data confirm that exosomes have a strong impact on the tumor initiation, progression, chemoresistance and metastasis of CRC (Figure 1). The application of exosomes as a novel diagnostic and therapeutic strategy have been confirmed by multiple researches in cell lines, animal models and human body fluids. However, the clinical utilities of the mentioned exosomal miRNA or proteins as biomarkers in diagnosis or prognosis of CRC patients require large, randomized clinical trials for further validation. Moreover, it remains unexplored whether exosomal biomarkers allow an earlier detection of CRC than other existing markers. Similarly, further translational studies and carefully designed clinical trials are essential to prove the therapeutic values of exosomes as delivery vector, exosome-based immunotherapy, or exosomes as target in CRC. These approaches could be used alone or in combination with established regimes such as chemotherapy, to treat CRC. They represent promising therapeutic opportunities especially for patients with metastatic CRC.
